# Correction: SPAG6 hypermethylation silences a novel tumor suppressor and inhibits renal cell carcinoma progression via PI3K/AKT/mTOR pathway

**DOI:** 10.1371/journal.pone.0345024

**Published:** 2026-03-13

**Authors:** Tianyu Wu, Xing Ji, Yongyang Yun, Xiaofei Wang, Ying Gan, Yu Fan, Qian Zhang

There are errors in the author affiliations. The correct affiliations are as follows:

Tianyu Wu ^1,2,3,4^, Xing Ji ^1,2,3,4^, Yongyang Yun ^1,2,3,4^, Xiaofei Wang ^5^, Ying Gan ^1,2,3,4^, Yu Fan ^1,2,3,4^, Qian Zhang ^1,2,3,4,6^

**1** Department of Urology, Peking University First Hospital, Beijing, China, **2** Institution of Urology, Peking University, Beijing, China, **3** Beijing Key Laboratory of Urogenital Diseases (Male) Molecular Diagnosis and Treatment Center, Beijing, China, **4** National Urological Cancer Center, Beijing, China, **5** Department of General Surgery, Xuanwu Hospital, Capital Medical University, Beijing, China, **6** Department of Urology, Beijing Shijitan Hospital, Capital Medical University, Beijing, China
